# Twitter Sentiment Analysis of Long COVID Syndrome

**DOI:** 10.7759/cureus.25901

**Published:** 2022-06-13

**Authors:** Toluwalase Awoyemi, Ujunwa Ebili, Abiola Olusanya, Kayode E Ogunniyi, Adedolapo V Adejumo

**Affiliations:** 1 Nuffield Department of Women's and Reproductive Health, University of Oxford, Oxford, GBR; 2 Family Medicine, Emel Hospital, Lagos, NGA; 3 Department of Haematology, University Hospitals Coventry and Warwickshire, Coventry, GBR; 4 Internal Medicine, University Hospital of North Durham, Durham, GBR; 5 Intensive Care Unit, Chelsea and Westminster NHS Foundation Trust, London, GBR

**Keywords:** health informatics, twitter, sentiment analysis, long haulers, long covid syndrome

## Abstract

Background

Long COVID syndrome originated as a patient phrased terminology which was initially used to describe a group of vague symptoms that persisted after recovering from COVID-19. However, it has moved from a patient *lingo* to a recognized pathological entity which refers to a group of symptoms that lasts weeks or months after the COVID-19 illness. The novelty of this condition, the inadequacy of research on long COVID syndrome, and its origin as a patient-coined terminology necessitated exploring the disease's sentiments and conversations by analyzing publicly available tweets.

Method

Tweets were extracted using the Twarc2 tool for tweets in the English language with the keywords (*long COVID syndrome, long COVID, post-COVID syndrome, post-acute sequelae of SARS-CoV-2, long-term COVID, long haulers, and chronic COVID syndrome*) between March 25, 2022, and April 1, 2022. The analyses included frequency of the keywords, sentiment analysis, and topic modeling to identify and explore discussion topics over time. A natural language approach and the latent Dirichlet allocation algorithm were used to determine the most shared tweet topics, categorize clusters, and identify themes based on keyword analysis.

Results

The search yielded 62,232 tweets. The tweets were reduced to 10,670 tweets after removing the duplicates. The vast majority of the tweets originated from the United States of America (38%), United Kingdom (30%), and Canada (13%), with the most common hashtags being #longcovid (36%) and #covid (6.36%), and the most frequently used word being people (1.05%). The top three emotions detected by our analysis were trust (11.68%), fear (11.26%), and sadness (9.76%). The sentiment analysis results showed that people have comparable levels of positivity (19.90%) and negativity (18.39%) towards long COVID.

Conclusions

Our analysis revealed comparable sentiments about long COVID syndrome, albeit slightly positive. Most tweets connoted trust (positive), fear (negative), and sadness (negative). These emotions were linked with concerns about the *infection, pandemic, chronic disability*, and *governmental policies*. We believe this study would be important in guiding information dissemination and governmental policy implementation necessary in tackling long COVID syndrome.

## Introduction

COVID-19 has ravaged health systems for the past two years since the first case was reported on December 31, 2019 [1]. Most conversations and scientific discussions around the virus and pandemic focused on the immediate and short-term pathogenic effects and disease. However, over the ensuing months and years, it became apparent that some COVID-19 symptoms persisted beyond the acute respiratory phase into a multisystemic condition. The trajectory of long COVID changed in October 2021, when the World Health Organization (WHO) released its definition of long COVID syndrome [2]. This was seventeen months after the hashtag #LongCovid first appeared on Twitter [3]. This singular act represented substantial progress that had been previously riddled with disbelief by some health practitioners and people who had not experienced the symptoms [4]. Since then, the multisystemic manifestations of long COVID syndrome have been published in peer-reviewed journals and gained wider acceptance in the scientific community [5-7]. Around twenty-one percent of patients with long COVID believed that medical staff were not attentive to them and were often dismissive of their complaints [8] or misdiagnosed. Roughly about 35%, 21%, and 10% of COVID-19 positive patients experienced symptoms that lasted longer than three, five, and 12 weeks respectively [7,9-11]. Most of these patients experienced fatigue or muscle weakness, sleep disorders, shortness of breath, anxiety, loss of taste and smell, cognitive disorders such as memory loss, difficulty concentrating, or depression [12,13]. Worse still, 30% of the patients experiencing this phenomenon were rehospitalized within five months, and 12% died [14].

This phenomenon is now referred to as long COVID syndrome, a term first coined as a hashtag on Twitter by Elisa Perego [15,16] after online conversations between patients who found each other and formed supportive groups on Twitter. This interactive social media platform allows users to send 140-character messages to one another: "long COVID" became universally adapted among patients, media channels, and organized clinical and policy channels in just a few months [16,17]. While the name has been refined since then to include post-COVID-19 condition, post-COVID-19 syndrome or chronic COVID syndrome (CCS), and post-acute sequelae of COVID-19 (PASC) [18-21]. A universal disease definition is still lacking, but many organizations have published case definitions that vary by country. The WHO defined long COVID as a post-COVID-19 condition that occurs in individuals with a history of probable or confirmed SARS-CoV-2 infection, usually three months from the onset, with symptoms that last for at least two months and cannot be explained by an alternative diagnosis [18], while The National Institute for Health and Care Excellence (NICE) describes "post COVID-19 syndrome" or "long COVID" as a set of persistent physical, cognitive, and/or psychological symptoms that continue for more than 12 weeks after illness and which are not explained by an alternative diagnosis [22]. Long COVID has a worse outcome in females, people with obesity, pre-existing psychiatric complications, and older age groups [23,24].

Anecdotal experiences have been shared on social media, traditional media, and individual patient groups, including infected doctors [25]. But very few studies have analyzed these publicly shared experiences to understand the public's perception and sentiments about long COVID syndrome. In this study, we explored the themes, public opinions, and sentiments regarding long COVID syndrome by analyzing Twitter data between March 25, 2022, and April 1, 2022, to understand the public perception and conversation surrounding long-term complications of long COVID syndrome. 

## Materials and methods

This study employed two data preparation steps: (1) data extraction using the application programming interface (API) for Twitter to collect tweets, and (2) data cleaning, filtering, and analysis to answer our research question.

Data collection

We collected Twitter data by connecting to Twitter using the Twitter streaming API. To access the Twitter API, we applied for access and developed an application for research access to generate the keys and tokens to extract and retrieve tweets. The data extraction phase was performed on PowerShell with the Twarc2 tool. We configured Twarc2 with our keys and token and searched for tweets between March 25, 2022, and April 1, 2022, using specifying keywords (*long COVID syndrome, long COVID, post-COVID syndrome, post-acute sequelae of SARS-CoV-2, long-term COVID, long haulers, and chronic COVID syndrome*) and focused on only tweets in the English language. The duration was chosen arbitrarily and shortened to eight days preceding the commencement of the study because of the enormous amount of data and the limitation in the analytic power available. The tweet search results were saved as JSONL files, flattened, and converted into a comma-delimited file (comma-separated values [CSV]) with twarc2. The converted file was then imported into R (version 4.1.2, R Foundation for Statistical Computing, Vienna, Austria).

Data analysis

The data analysis was conducted using R Studio. We pre-processed the imported file (62,232 tweets) in R to include only English tweets. After this step, only 10,670 tweets were left. A custom R code was generated to clean and pre-process filtered tweets by removing URLs, emojis, special characters, retweets, numbers, graphs, digits, numbers, white spaces, symbols such as @./,// websites, and any identifiable information, hyperlinks and hash symbols.

The tweets were converted to a corpus (text mining structure) and then transformed to lower case to remove ambiguity from mixed case tweets. Known English stop words (e.g., for, the, is) were extracted using the *TM *package. Furthermore, we removed words such as 'corona' or 'virus' that may be unrelated to our topic of interest. 

After which, we created a document-term matrix and calculated the term frequency-inverse document frequency (TF-IDF), a metric of word importance in a corpus. We set out to answer the objective of this study in three connected ways. First, we analyzed the frequencies of single words (unigram) in the text mining structure and visualized the most common words with a bar plot. This type of content analysis enabled us to determine themes or terms most related to the public perception of long COVID symptoms. 

Second, we performed a sentiment analysis on the extracted tweets. Here, we used the *get_nrc_sentiment *from the *Syuhzet *package to obtain sentiments from eight different types of emotions (anger, anticipation, disgust, fear, joy, sadness, surprise, and trust) with the National Research Council (NRC) sentiment lexicon. Positive emotions were trust and joy, while anger, sadness, fear, and disgust were negative. Surprise and anticipation can be either negative or positive. The NRC lexicon has no neutral words; words that are not found in its classification system were excluded from the analysis.

Third, we identified the most common topics in the tweets to categorize clusters and find themes based on the keyword analysis using a topic modeling approach based on unsupervised machine learning analysis and the latent Dirichlet allocation (LDA) algorithm. LDA is an unsupervised document classification method like clustering on numeric data; it can be used to find natural groups of items even when it is not sure what is being searched. The LDA algorithm is a trendy method of model fitting. It treats each document as a combination of topics and each topic as a concoction of words. In this process, tweeted messages can overlap in content rather than be separated into different groups. We used the *topicmodels *package in R for topic modeling analysis. LDA is a parameterized model; thus, we had to predefine the number of topics K to input in advance. To estimate the optimal K, we used the *ldatuning* package and chose a number based on the results of two selection metrics (Griffiths2004, and Arun2010). *ldatuning *was set to run serially from two to 30 topics, and we found 28 to be the optimal number (see Appendix). We thus used 20 to generate our LDA model with the Gibbs algorithm. To ensure reproducibility, we set the K (number of topics) at 28, set the seed at 1234, and the alpha was set at 0.1. We repeated the analysis five times and obtained the same result each time. We presented the results of the topic models as bar charts.

Finally, to visualize the word network, we fragmented the tweets into words (tokenization) and counted the pairwise relative occurrence of words. We next filtered for stop words and removed white spaces as previously described. Finally, we grouped and counted by bigram. We visualized the bigram count network as a node map using the *network3D *package and set the weight threshold at 50. Each node represents a word, and two terms will be connected if they appear as a bigram (that is used together frequently in a tweet). These pairwise word counts provide additional information about the relations of the words in the extracted tweets.

## Results

Descriptive analysis of long COVID tweets

**Figure 1:**
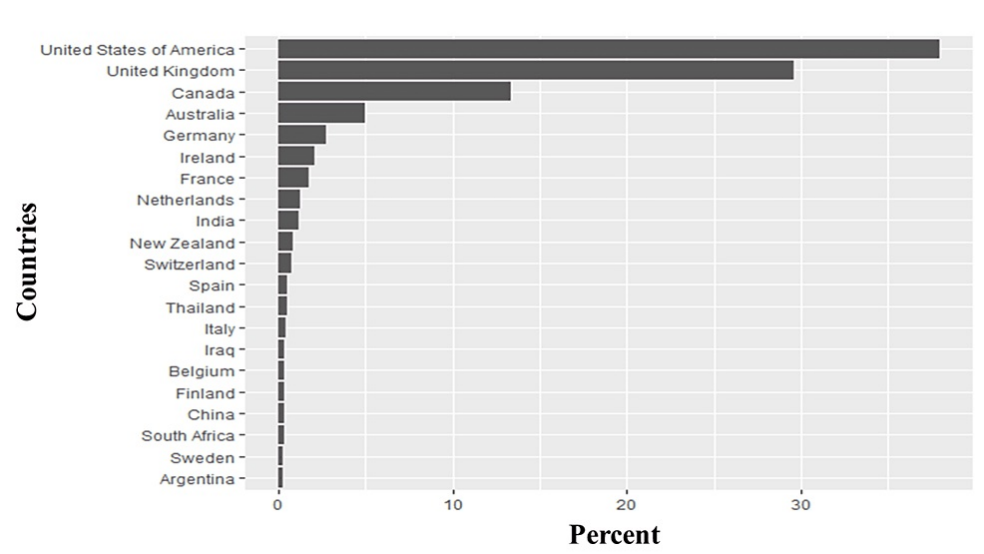
Countries of origin of tweets analyzed in this study The Y-axis contains the top 25 countries with the most 'long COVID syndrome' tweets, while the X-axis is the percent of each country relative to the whole tweet.

**Figure 2:**
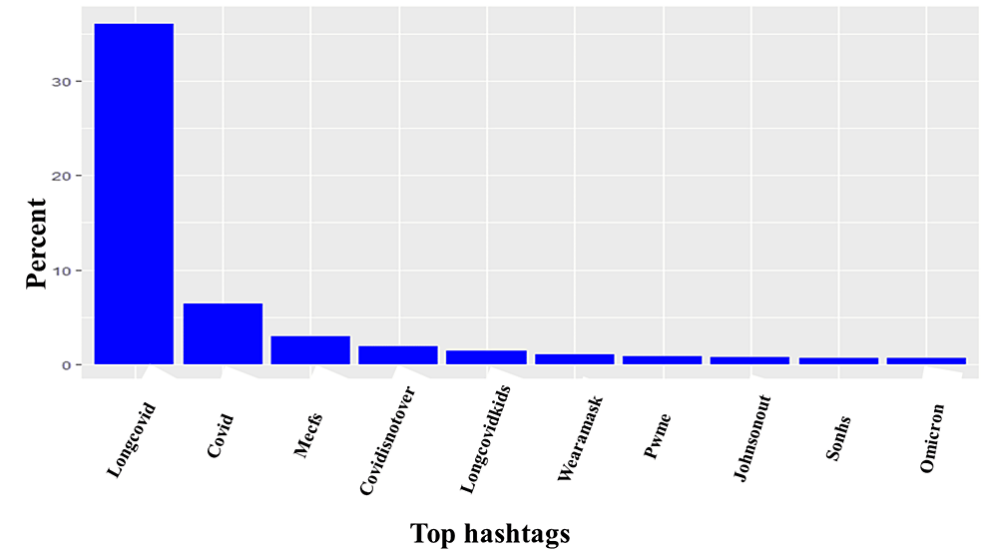
Top ten hashtags used in association with long COVID tweets The Y-axis is the percent of each hashtag relative to all analyzed tweets, while the X-axis contains the top ten hashtags used in association with long COVID tweets.

**Figure 3:**
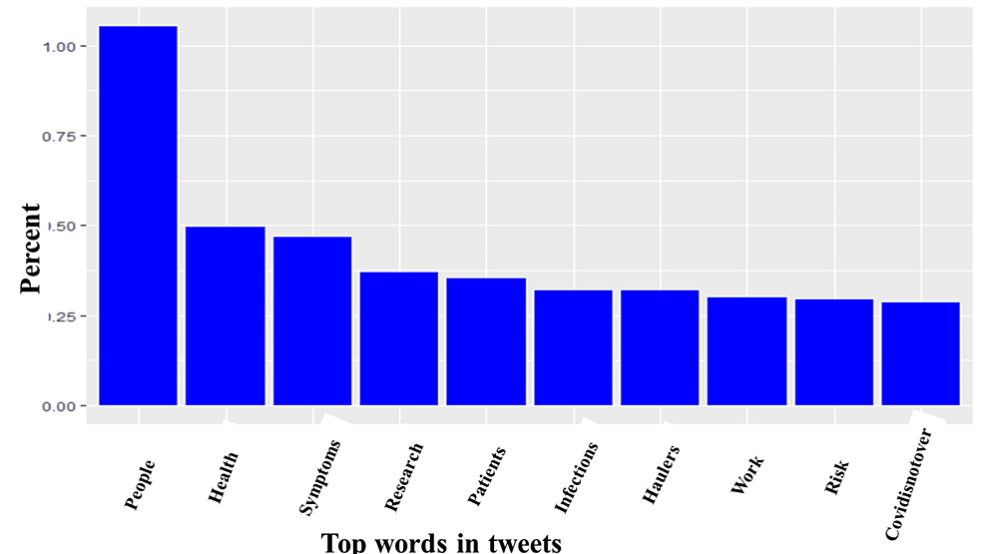
Top ten words used in long COVID tweets The Y-axis is the percent of each word relative to all analyzed tweets, while the X-axis contains the top ten words used in long COVID tweets.

**Figure 4:**
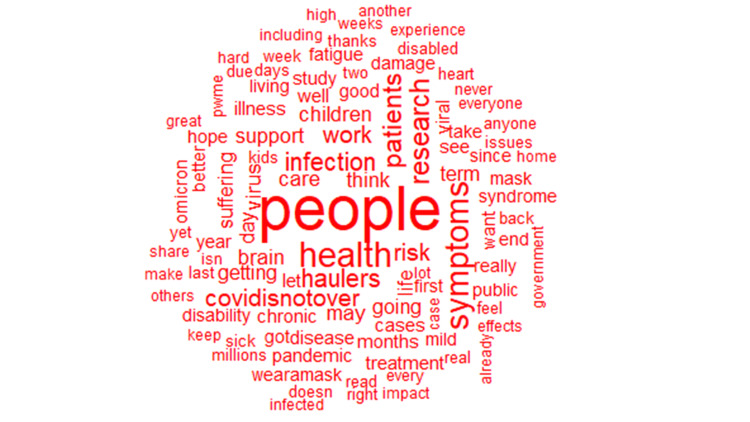
Word cloud of long COVID words with a frequency of occurrence greater than twenty-five

As discussed in the methods section, we analyzed 62,232 tweets. After removing duplicates, we had 10,670 tweets left. Of the tweets analyzed, the minimum number of likes per tweet was one, the maximum per tweet was 619, and the median likes per tweet was 113. Similarly, the minimum number of quotes per tweet was one, while the maximum number of quotes per tweet was 119, and the median number of quotes per tweet was two. Likewise, the minimum number of replies per tweet was one, while the maximum number per tweet was 137, and the median number per tweet was two. Finally, the lowest retweets per tweet was one, the maximum retweets per tweet was 367, and the median retweets per tweet was two. Almost half (44.13%) of the tweets had no location. However, of those where the location of the tweet could be obtained, ninety-six countries were represented, and the vast majority originated from the United States of America (38%), the United Kingdom (30%), and Canada (13%; Figure 1). After analyzing the tweets, we observed that the five most common hashtags were #longcovid (36%), #covid (6.36%), #mecfs (2.92%), #covidisnotover (1.87%), #longcovidkids (1.40%; Figure 2). Similarly, the five most frequently used words were people (1.05%), health (0.49%), symptoms (0.46%), research (0.37%), and patients (0.35%; Figures 3-4). Our analysis showed that *'strategy*' was most commonly tweeted with words like '*eradication,' 'suppression,' 'act,' and 'carelongcovid,' *while* 'indoor' *was associated with '*strict,' 'outdoor,' 'lockdowns,' 'eradication' and 'suppression' *(Table 1).

**Table 1:** Long COVID words and frequently correlated words (Pearson correlation coefficient greater than 0.60)

Word	Correlation coefficient	Word	Correlation coefficient	Word	Correlation coefficient
Strategy		Total		Necessary	
Eradication	0.67	Eradication	0.76	Strict	0.76
Suppression	0.67	Suppression	0.74	Outdoors	0.75
Act		Strict	0.68	Lockdowns	0.73
Careforlongcovid	0.67	Outdoors	0.67	Eradication	0.69
Indoor		Lockdowns	0.65	Suppression	0.68
Strict	0.76	Mandates			
Outdoors	0.75	Lockdowns	0.67		
Lockdowns	0.73	Strict	0.67		
Eradication	0.69	Outdoors	0.66		
Suppression	0.68	Eradication	0.61		

Sentiment and emotional quotient analysis of long COVID tweets

**Figure 5:**
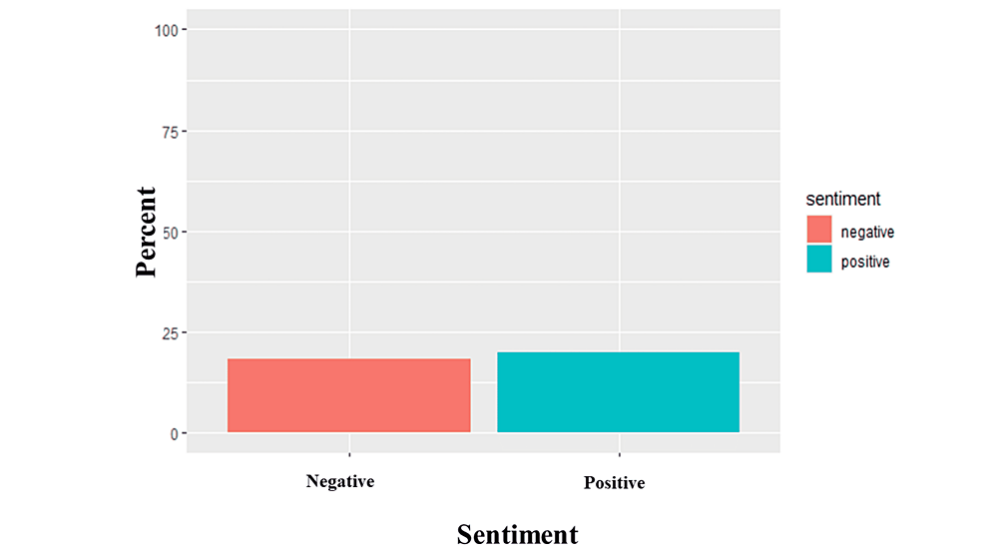
Sentiment analysis of long COVID tweets The Y-axis is the percent of sentiments of all analyzed tweets, while the X-axis refers to the sentiments (negative or positive) in long COVID tweets.

**Figure 6:**
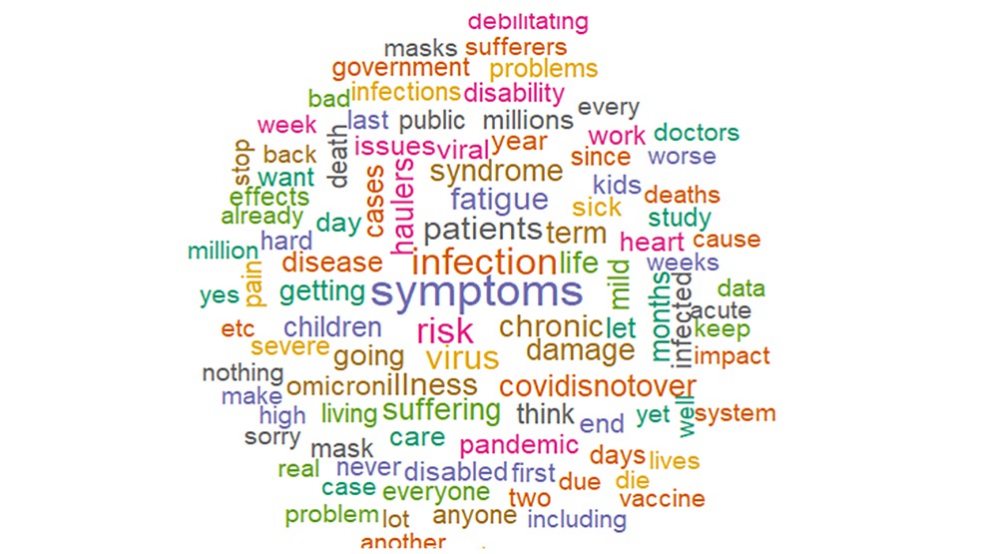
Word cloud of the top 100 negative long COVID words

**Figure 7:**
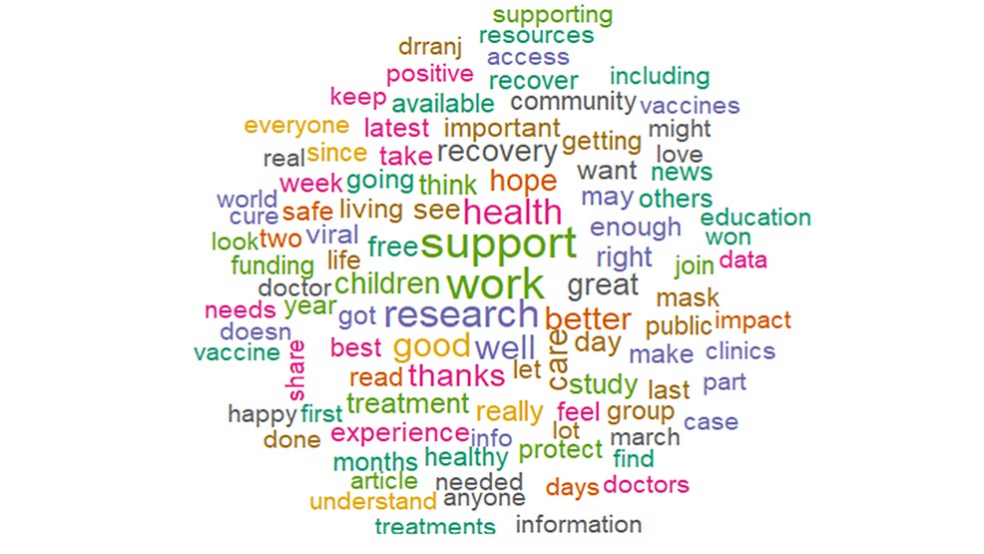
Word cloud of the top 100 positive long COVID words

**Figure 8:**
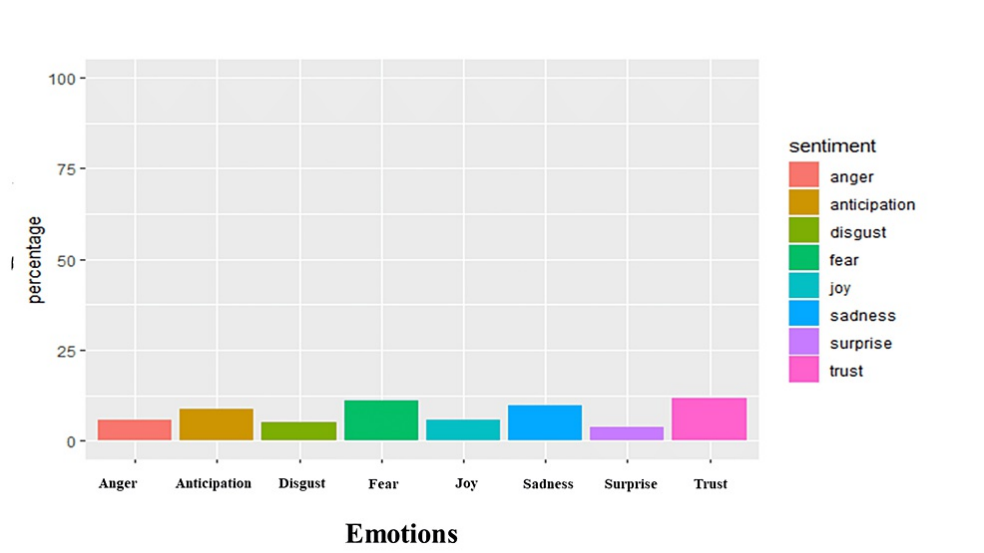
Emotion quotient analysis of long COVID tweets The Y-axis is the percent of sentiments of all analyzed tweets, while the X-axis refers to the emotion in long COVID tweets.

**Figure 9:**
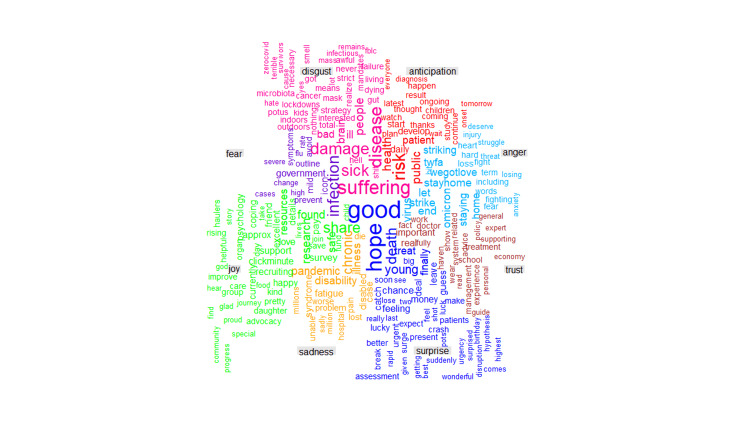
Comparative word cloud of the long COVID words and portrayed emotions

There were similar levels of positivity (19.90%) and negativity (18.39%) expressed in the analyzed tweets (Figure 5). The analysis showed the expression of negative feelings through words such as *'symptoms,' 'infection,' 'risk,' 'haulers,'* and *'disease*' (Figure 6) and positive feelings through words like *'research,' 'support,' 'health,' 'recovery,'* and *'hope'* (Figure 7). Trust (11.68%) was the most abundant emotion, followed by fear (11.26%) and sadness (9.76%; Figure 8). Conversely, surprise (3.68%), disgust (5.16%), and joy (5.65%) were the least expressed emotions. Fear was about *'infection,' 'high,' 'mild,' 'government,' 'severe,' *and *'flu' *(Figure 9), and sadness was used in the context of *'pandemic,' 'syndrome,' 'chronic,' 'disability,' a*nd* 'illness.' *Likewise, *'stayathome,' 'omicron,' 'virus,' 'strike,' *and* 'end'* denoted anger and *'suffering,' 'sick,' 'hell,' 'damage' a*nd* 'brain' *showed disgust. For the positive emotions, trust was used about *'doctor,' 'important,' 'fact,' 'policy,' 'haven,'* and *'treatment,'* and *'research,' 'resources,' 'friend,' 'excellent,' 'safe,'* and *'share'* tweeted in the context of joy. Finally, *'risk,' 'health,' 'public,' 'daily,' 'plan,'* and *'develop'* reflected anticipation and *'hope,' 'death,' 'young,' 'finally,'* and *'good'* represented surprise.

Topic modeling and network analysis of long COVID tweets

**Figure 10:**
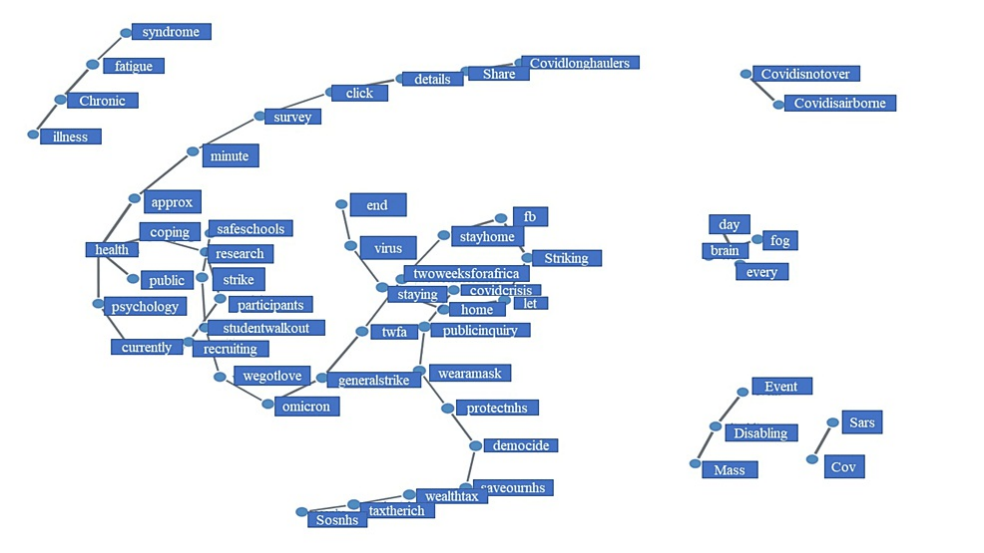
A network analysis of common co-occurring long COVID words The nodes represent words while the edges (lines) are the connection's weight between the nodes. The thicker the lines, the stronger the connection.

Our analysis reveals that *'illness,' 'chronic,' 'fatigue,'* and* 'syndrome' *were commonly tweeted together and may represent symptoms, while* 'covidisnotover' *and *'covidisairborne' *were tweeted commonly in the same context (Figure 10). The two largest networks included sixteen words each. The first contains words such as* 'covidlonghaulers,' 'share,' 'details,' 'click,' 'survey,' 'minute,' 'approx.,' 'psychology,' 'currently,' 'recruiting,' 'participants,' 'research,' 'coping,' 'health'* and '*public,' *while the second includes '*safeschool,' 'strike,' 'studentwalkout,' 'wegotlove,' 'omicron,' 'generalstrike,' 'twfa,' 'twoweeksforafrica,' 'stayhome,' 'fb,' 'striking,' 'let,' 'home,' 'staying,' 'virus,'* and '*end.'*

**Figure 11:**
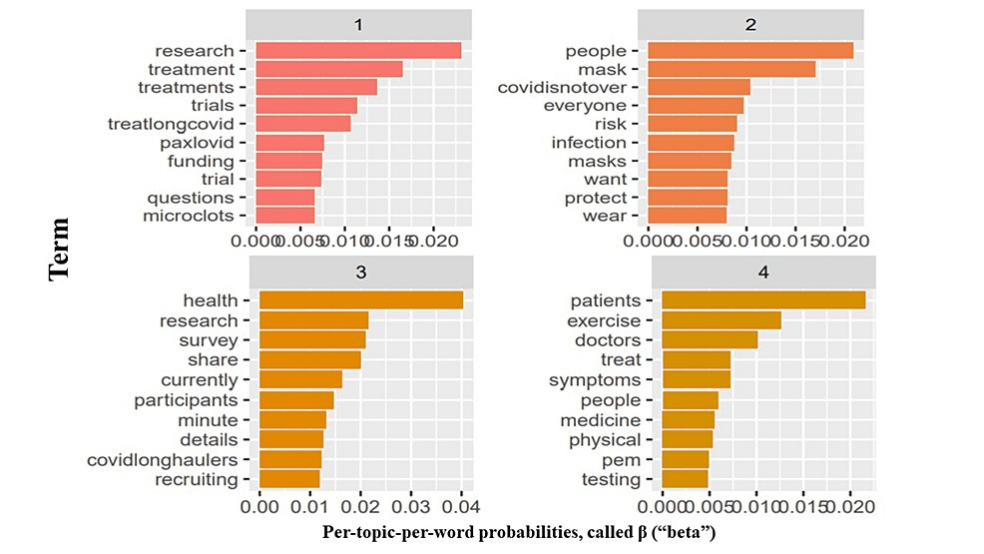
Modeled topics and their associated frequent terms among the analyzed long COVID syndrome tweets analyzed

Finally, we used the LDA topic modeling to identify four emergent topical themes and discourses regarding long COVID syndrome based on topic coherence (Figure 11). The topics give an overall idea of the nature of the conversation in the tweets analyzed. Topic one contained words such as *'research*,' *'treatment,' 'treatments,' 'trials,' 'treatlongcovid,' *and *'paxlovid,'* while for topic two, *'people,' 'mask,' 'covidisnotover,' 'everyone,' 'risks,' *and *'infection.'* Likewise for topic three, *'health,' 'research,' 'survey,' 'share,' 'currently,' *and *'participants,' *finally, topic four symptoms,* 'patients,' 'exercise,' 'doctor,' 'treat,' 'symptoms,' *and *'people.'*

## Discussion

Long COVID syndrome is yet to be fully characterized or understood. Still, its health impact is undoubtedly crippling and life-changing. It imposes physical limitation, financial hardship, disruption of social relationships, conflict of social roles, and social stigma on patients with long COVID syndrome [26]. The term "long haulers," which is how patients commonly refer to themselves, was coined by Amy Watson, a patient and convener of the 'long haul COVID fighters' [16]. It was initially considered anecdotal, with only patient lived experience narratives and no official or scientific data available. This disbelief is not unusual among chronic illnesses such as postural tachycardia syndrome (POTS), who are either misdiagnosed or have to wait for years for the right diagnosis to be made [27]. 

In this paper, we explored the themes, public opinions, and sentiments of Twitter users about long COVID syndrome. Almost half of Twitter users (44.13%) did not share their location, but of those who did, the vast majority of online conversations originated from the United States of America (38%), the United Kingdom (30%), and Canada (13%). This is similar to the demographics of online responses from a patient-led worldwide survey to assess the post-COVID 19 symptoms, where the US, UK, and Canada were in the top five countries assessed [8]. This finding may be reflective of our decision to specifically select 'English' hashtags in contrast to hashtags from other languages #apresJ20 (French), #covidpersistente (Spanish), #MitCoronaLeben (German), #koronaoire (Finnish); #長期微熱組 (Japanese) [16] and filtering to include only English tweets, but it may also indicate that the awareness of long COVID syndrome is poor outside of North America and some regions of Europe. Other important sociodemographic variables could not be analyzed because Twitter users do not routinely identify with sociodemographic information like gender and socioeconomic status.

We also found* that #mecfs, #covidisnotover, #longcovidkids, #wearamask, #pwme, #johnsonout, and #sonhs* were the commonly used hashtags in addition to the popular COVID-19-related hashtags *(#omicron, #longcovid, and #covid*), *#covidisnotover *and *#wearamask* were used to draw attention to the rising cases of COVID, the seriousness of long COVID, and the continuous need to keep using masks. #*pwme* and *#mecfs *(myalgic encephalomyelitis [ME]/chronic fatigue syndrome [CFS]) were acronyms that referenced people with ME/CFS and drew comparisons between other chronic fatigue and long COVID syndromes.* #longcovidkids* depicts the growing concern of Twitter users about children experiencing long COVID symptoms.

The five most frequently used words were* 'people,' 'health,' 'symptoms,' 'research,' * and '*patients,'* with* 'people'* being the most used word. These words highlighted symptoms of long COVID, patient experience, the impact of long COVID on health, and the need for research on long COVID and other chronic illnesses like ME/CFS. Our analysis showed that* 'strategy' *was most commonly co-tweeted with '*eradication,' 'suppression,' 'act,'* and *'carelongcovid,' *while *'indoor' *was co-tweeted with *'strict,' outdoor,' 'lockdowns,' 'eradication'* and* 'suppression*.' Identified co-tweeted words were in reference to preventive strategies necessary to suppress the spread of COVID-19 or potentially eradicate the virus from circulation.

We observed that the tweets analyzed in this study were slightly positive with words like *'research,' 'support,' 'health,' 'recovery,' *and *'hope.' *This is different from the study by Miyake et al. who reported that the sentiment towards long COVID, particularly in the first wave, was overwhelmingly negative (22%) compared to positive (7%) [28]. However, our research was conducted during the third wave, not the first wave, and may be reflective of the increasing knowledge and acceptance of long COVID, increased availability or support and research (as exemplified by the keywords of positive sentiments -* 'research,' 'support,' 'health,' 'recovery,'* and '*hope'*) into long COVID over the ensuing two years. This is further buttressed by '*trust' *being the most prominent emotion in our study, particularly in relation to* 'doctor,' 'important,' 'fact,' 'policy,' 'haven,'* and '*treatment' *commonly used. This likely reflects the acceptance and recognition of long COVID by health authorities; World Health Organization (WHO), National Institute for Health and Care Excellence (NICE), Centers for Disease Control and Prevention (CDC), and the US National Institutes of Health (NIH) through the publication of official case definitions [18,21,29]. In addition, different countries (UK, US, and Australia) have set up specialized facilities for the treatment and management of long COVID or allocated money and resources to support research on long COVID syndrome [10,29-32]. 

In contrast, the negative sentiments were slightly less, with words like *'symptoms,' 'infection,' 'risk,' 'haulers,' *and *'disease'* more frequently used. We noted that the most prominent negative emotions were fear due to '*high infection rate,' 'mild (symptoms),' '(poor response from the) government,' 'severe (symptoms)'* and 'f*lu,'* and sadness about the '*pandemic,' 'syndrome,' 'chronic/chronicity of the long COVID,' 'disability,' a*nd* 'illness.' * A patient-led survey similarly noted fear in their survey results, though for a different reason. The fear in their study was attributed to being stigmatized at work, protecting privacy, avoiding stigma, misleading with false information (due to lack of testing), or being blamed for enabling the spreading virus [8,33]. Similarly, Aghaei et al. noted that social isolation, COVID-19-associated stigma, and conflicts of social roles cause tremendous stress to women with long COVID syndrome [26]. This difference might be due to the progress made since both of those studies were conducted, as awareness about long COVID is rapidly increasing. We decided not to stratify the sentiments by location because only a proportion of the tweets (55.87%) reported a location. In addition, as said in the methods, the NRC lexicon does not contain a neutral class, and though it is very likely that words may have a neutral connotation, if such words are not present in any of the NRC lexicon classifications, they would be automatically excluded by the algorithm. It is also unlikely for tweets to be both positive and negative.

Finally, our analysis identified four common topics. Topic one roughly talks about the treatment of long COVID patients, topic two reinforces the protection of those at risk by the continued practice of COVID preventive measures, and topic three draws attention to ongoing research and surveys about long COVID syndrome. Topic four may imply the management modalities of long COVID syndrome. We also showed that '*illness,' 'chronic,' 'fatigue,' *and *'syndrome' *were commonly tweeted together and may represent symptoms, while* 'covidisnotover' *and* 'covidisairborne'* were tweeted commonly in the same context. However, there are some limitations; we focused on tweets in English to prevent the loss of information while translating. We also could not analyze more tweets due to computational limitations imposed by analyzing massive data. In addition, the field of lexicon-based sentiment analysis is still developing; it is difficult to address tweets from trolls, sarcastic or bi-emotional tweets.

## Conclusions

Long COVID syndrome is perhaps the first medical condition to have originated from online patient communities and support groups. We were able to identify thousands of Twitter data that provided unique insights into the experiences of patients with long COVID syndrome. Our sentiment analysis evaluated long COVID sentiments against the backdrop of waning governmental COVID 19 restrictions and guidelines. We strongly believe our results are interesting and informative. We observed that the levels of positive and negative sentiments are quite similar, though the sentiment is still mainly positive. The most dominant emotions are trust (positive), fear, and sadness (negative). We observed that patients are starting to develop confidence in medical practitioners, policies, and treatment. This is reflected by the predominance of trust as the most observed emotion in the analyzed tweets. However, we had some limitations; we focused on tweets in English to prevent the loss of information while translating. We limited our analysis to less than 15,000 tweets due to computational and storage space limitations. We hope our study would help inform and direct medical practitioners, governmental and non-governmental organizations, friends, families, and caregivers of patients with long COVID syndrome on the need to come up with creative approaches to solidify and justify the trust, help address the fear and sadness faced by patients.

## Figures and Tables

**Figure 12:**
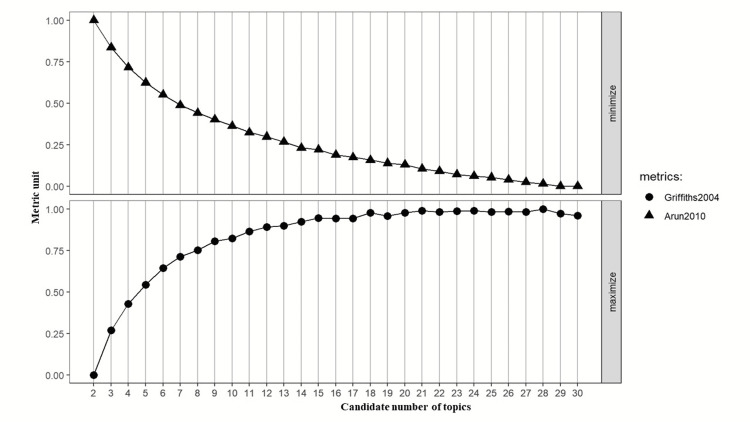
Result of LDA tuning using "Griffiths2004" and "Arun2010" metrics The Y-axis contains the metric unit while the X-axis contains the candidate number of topics. We identified topic numbers 28-30 (Griffith) and 20-30 (Arun) to be the optimal range. Based on both results, we set our K (topic number) at 28. LDA - latent Dirichlet allocation
